# Endothelial thrombomodulin—Its role in trauma‐induced coagulopathy

**DOI:** 10.1111/trf.70089

**Published:** 2026-01-29

**Authors:** Jeries Abu‐Hanna, Gael B. Morrow, Gang Xu, Lewis Timms, Naveed Akbar, Michael Laffan, Robin P. Choudhury, Nicola Curry

**Affiliations:** ^1^ Oxford Haemophilia & Thrombosis Centre Oxford University Hospitals NHS Foundation Trust Oxford UK; ^2^ Radcliffe Department of Medicine Oxford University Oxford UK; ^3^ School of Pharmacy and Life Sciences Robert Gordon University Aberdeen UK; ^4^ Division of Cardiovascular Medicine Oxford University Hospitals NHS Foundation Trust Oxford UK; ^5^ Department of Immunology and Inflammation, Faculty of Medicine, Imperial College London, University of London London UK

**Keywords:** hemostasis, endothelial colony forming cells, fibrinolysis, thrombomodulin, trauma‐induced coagulopathy

## Abstract

**Background:**

Trauma‐induced coagulopathy (TIC) describes a complex set of coagulation changes affecting severely injured patients. The thrombomodulin‐protein C axis is central to the evolution of TIC. Soluble thrombomodulin (sTM) levels are elevated after injury and predict poor clinical outcomes. Recently, a heritable bleeding disorder defined by injury‐related excessive bleeding, markedly elevated sTM and a *THBD* mutation has been described. The clinical phenotype broadly mirrors that of trauma hemorrhage, and we hypothesized that their endothelial colony forming cells (ECFCs) could be used to model trauma‐induced endothelial damage.

**Study Design and Methods:**

Our objectives were to: (a) define the hemostatic capacity of wild type (WT‐) and *THBD* variant (MT‐) ECFCs, using global hemostatic assays, (b) determine the hemostatic changes on ECFC surfaces after exposing cells to trauma stimuli, (c) evaluate the contributions of traumatized ECFCs and trauma patient plasma to overall hemostatic capacity, to better understand the injured endothelial‐coagulation interface.

**Results:**

We show that trauma stimuli cause significant, rapid shedding of TM from WT‐cells and this reduced membrane‐TM supports quicker fibrinolysis and greater thrombin generating capacity. Combining trauma plasma with traumatized ECFCs attenuates and/or negates these effects.

**Conclusion:**

This ECFC model provides novel mechanistic insights into TIC at the endothelial surface, informing future treatment strategies.

## 1 INTRODUCTION

Globally 1.2 million people die from uncontrolled bleeding following traumatic injury every year.^1^ The risk of severe hemorrhage is highly dependent on whether a patient develops an associated clotting abnormality, called “trauma‐induced coagulopathy” (TIC).^2^ Research investigating the pathobiology of TIC has focused on evaluating temporal changes to clotting factor levels and correlating these with clinical outcomes.^2^, ^3^, ^4^ More recently, evidence has linked systemic endothelial damage, defined by high levels of circulating biomarkers such as thrombomodulin (TM) with TIC.^2^, ^5^ There is limited mechanistic research evaluating the interactions between the endothelium and the coagulation system in trauma hemorrhage.^5^ Such an approach may provide insights into this complex coagulopathy.

Thrombomodulin (TM), encoded by *THBD*, is a transmembrane glycoprotein expressed on endothelial cells (ECs) and it is one of the most important proteins orchestrating normal hemostasis at the endothelial‐coagulation interface.^6^, ^7^ TM binds avidly to thrombin, and the resulting thrombin‐TM complex activates protein C and thrombin activatable fibrinolysis inhibitor (TAFI), generating activated PC (aPC) and TAFI (TAFIa), with anticoagulant and antifibrinolytic effects, respectively.^6^, ^7^ In health, TM activity serves to restrict clot formation to the site of injury whilst promoting clot stability. Several lines of evidence have implicated TM as a key protein linking trauma‐induced endotheliopathy and coagulopathy. Not only are soluble TM (sTM) levels elevated in the blood of all injured patients, but also higher levels are further associated with severe bleeding and TIC^8^, ^9^, ^10^, ^11^ and rise in parallel with other markers of endothelial damage.^10^, ^11^, ^12^ Added to this, is the recent discovery of a heritable bleeding condition caused by variants in the *THBD* gene.^7^, ^13^, ^14^, ^15^, ^16^, ^17^ Affected patients have elevated sTM levels—up to 80‐fold normal—and report excessive bleeding after injurious events, for example, surgery.^15^, ^16^ The high sTM has two opposing effects in plasma: attenuating thrombin generation and delaying fibrinolysis.^15^ The hemostatic effects at the endothelial cell surface, which would be predicted to oppose those seen in plasma, have not been explored. The clinical phenotype of patients with *THBD* variants, coupled with reduced endothelial‐bound TM and high sTM, broadly reflect characteristics seen after trauma, leading us to hypothesize that their endothelial colony forming cells (ECFCs) may provide an innovative tool to aid the understanding of the role of TM in TIC.

Here, we use ECFCs,^18^, ^19^ cultured from healthy volunteers and patients with a heterozygote *THBD* variant^20^ to investigate how changes to endothelial bound TM impact TIC and trauma‐induced‐endotheliopathy.^18^ Our primary aims were to: (a) define and compare the hemostatic capacity of wild type ECFCs and ECFCs with the *THBD* variant using global hemostasis tests, thrombin generation (TG) and clot lysis (CL), (b) determine the hemostatic effects at the ECFC surfaces after exposing the cells to trauma stimuli over a 24‐h period and (c) evaluate the overall hemostatic outcomes when combining trauma‐stimulated ECFCs with plasma collected from trauma patients to better understand the endothelial‐coagulation interface early after injury.

## 2 METHODS

### 2.1 Trauma patients

Adult patients who met criteria for trauma team activation and arrived within 2 h of injury were eligible. Details of the Activation of Coagulation and Inflammation in Trauma (ACIT) study have been published.^21^ The study was approved by East London Regional Ethics Committee: 07/Q0603/29 and included approval for the enrollment of participants without personal, informed consent. Up to 20 mL blood was drawn within 20 min of admission and centrifuged (3000 × *g*, room temperature, 20 min) to obtain platelet poor plasma (PPP) and stored at −80°C until analyzed. Routine, clinically relevant blood samples were also taken, including full blood count, coagulation screen, Clauss fibrinogen, and D‐dimer. Patients chosen for this study had not received tranexamic acid prior to blood sample draw. Informed written consent was gained as soon as clinically possible, and where a participant did not regain capacity, personal or professional written assent was sought.

### 2.2 Patients with THBD gene variant and healthy volunteers

Ethical approval was granted by Wales Research Ethics Committee: 20/WA/0313. Written informed consent was sought prior to study inclusion and blood draw for all participants.

Additional, more detailed information about the cell culture methods and the hemostatic assays used can be found in Data S1, Supporting Information.

### 2.3 Cell culture

Endothelial cell colony forming cell (ECFC) cultures were established from five donors with wildtype TM (WT‐TM) and two patients with heterozygous TM variant p.Pro496Argfs*10 (MT‐TM, Table S1) as previously described.^22^, ^23^, ^24^, ^25^ For clot lysis, thrombin generation, and zymogen activation assays, ECFCs were seeded in 96‐well plates at 10,000 cells/well, incubated for 48 h. For supernatant collection, ECFCs were seeded in 6‐well plates at 100,000 cells/well, grown to confluence.

### 2.4 Cell treatment

To recapitulate traumatic injury, ECFCs were treated with trauma‐related stimuli optimized in our laboratory: 1 nM epinephrine (Sigma‐Aldrich), 0.1 ng/mL TNF‐α (R&D Systems), 0.1 ng/mL interleukin‐6 (IL‐6) (R&D Systems), 500 ng/mL high mobility group box 1 (HMGB1) (Abcam), 10 μM H_2_O_2_, incubated for 2 or 24 h, 1% O_2_. Control conditions: phosphate buffered saline (PBS), humidified CO_2_ incubator, ~20% O_2_.^20^

### 2.5 Flow cytometry

ECFCs were stained using fluorophore‐conjugated antibodies (Table S2). Specificity was confirmed using isotype controls (Figure S1). Ten thousand events were acquired on a BD LSR Fortessa X‐20 Cell Analyzer (BD Biosciences). Data were analyzed using FlowJo software (v10.10).

### 2.6 Immunocytochemistry

ECFCs were fixed with 4% paraformaldehyde, permeabilized with 0.1% Triton X‐100, blocked with 10% normal goat serum, incubated overnight at 4°C. Cells were subsequently washed with PBS and incubated with secondary antibodies (Table S3) and Alexa Fluor 647‐Phalloidin (Invitrogen; 1:300) for 1 h, room temperature, mounted with ProLong Gold Antifade Mountant with nuclear stain DAPI (Invitrogen), imaged using a Leica TCS SP8 confocal laser microscope, 40× magnification.

### 2.7 ELISA

Supernatants collected from ECFCs, PPP from trauma patients and healthy volunteers^8^ were assayed by ELISA (all commercial kits from AbCam, UK, unless stated). Thrombomodulin, factors V and VIII, plasmin‐antiplasmin (PAP, Technozym, USA), activated protein C, APC (2b Scientific Ltd., UK), plasminogen activator inhibitor‐1, PAI‐1, tissue‐plasminogen activator, tPA, and fibrinogen were quantified in PPP. Absorbance was read according to manufacturer instructions, using the Multiskan Ascent microplate reader (ThermoScientific) and the Ascent Software (v2.6).

### 2.8 Clot lysis

Clot lysis (CL) was performed on ECFC surfaces using pooled normal plasma (PNP; PrecisionBiologic), PPP from trauma patients, thrombin‐activatable fibrinolysis inhibitor, TAFI‐deficient plasma (TAFI‐DP; Affinity Biologicals), and with 50 pM tPA. Clotting was initiated with 0.1 U/mL thrombin (Sigma‐Aldrich), 10.6 mM calcium chloride, CaCl_2_ (Sigma‐Aldrich). Clot formation and lysis were monitored with absorbance (405 nm) every minute for 4 h, analyzed using Shiny App software.^26^

### 2.9 Thrombin generation

Thrombin generation (TG) was measured using the calibrated automated thrombography (CAT) method.^27^ TG was triggered with Diagnostica Stago PPP‐Low (tissue factor (TF) 1 pM + phospholipid 4 μM), thrombin fluorogenic substrate (Z‐Gly‐Gly‐Arg‐AMC), and CaCl_2_ (Diagnostica Stago, France). In some experiments, PNP, PPP from trauma patients, and protein C‐deficient plasma (PC‐DP; Affinity Biologicals) were analyzed.

### 2.10 Zymogen activation

Protein C (PC) activation was assessed using 70 nM PC from human plasma (Sigma‐Aldrich).^28^ Absorbance at 405 nm was measured every 30 s for 2 h.

### 2.11 Data analysis

All statistical analyses were performed on GraphPad Prism (v10.3.1). Normality was assessed using visual histogram assessment and Shapiro–Wilk test. Results were represented by mean ± standard deviation (SD)/median ± interquartile range (IQR), comparisons made using *t* tests or Mann–Whitney, as appropriate. Wilcoxon matched‐pair signed rank test compared matched groups. Normal ranges were calculated from 20 healthy volunteers and 1.96 SD of the mean or log‐transformed mean.^29^ Significance was set at *p* < 0.05.

## 3 RESULTS

### 3.1 MT‐TM ECFCs exhibit reduced surface expression of thrombomodulin which permits greater fibrinolysis and thrombin generation at the endothelial surface

**FIGURE 1:**
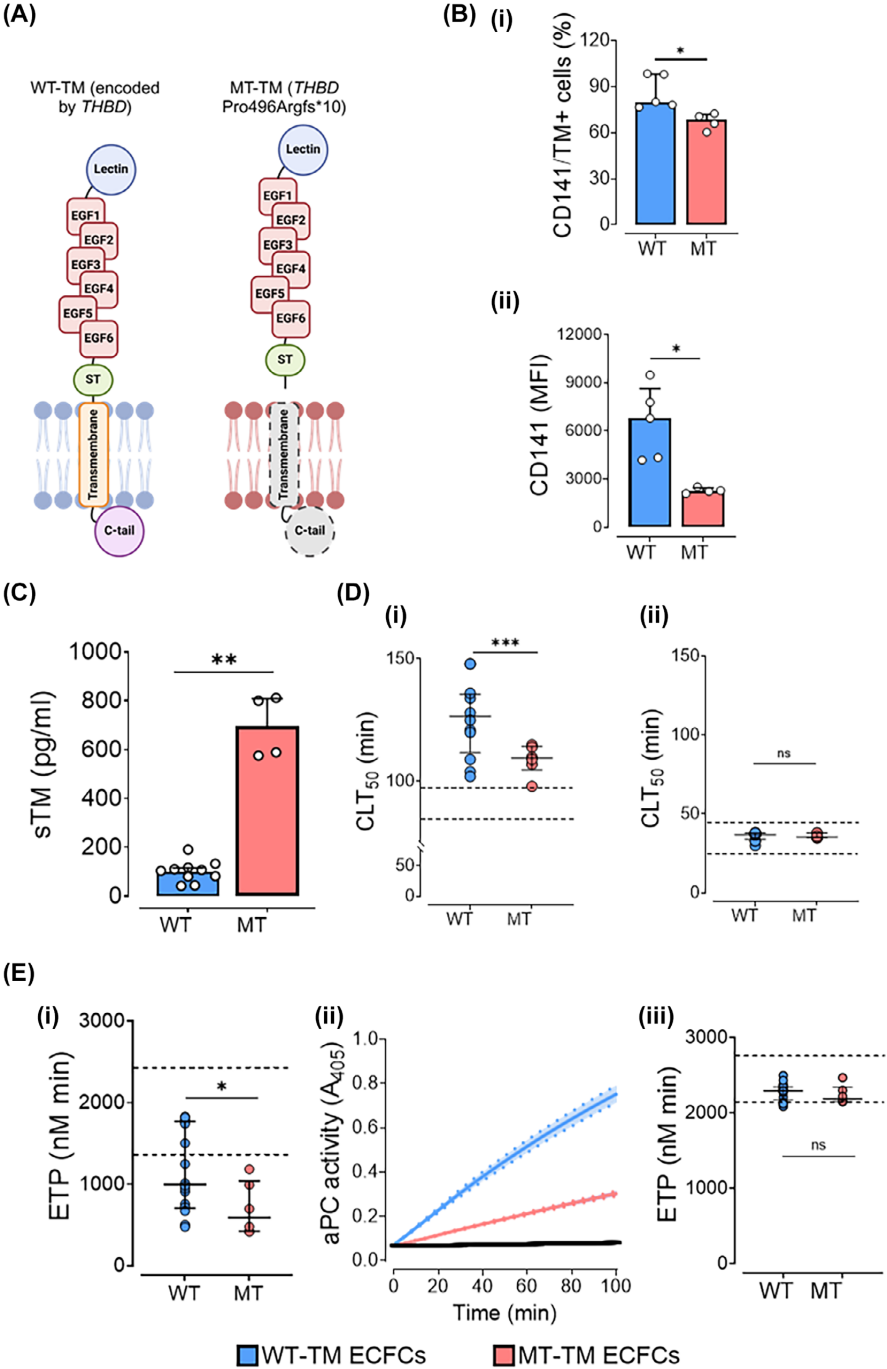
Hemostatic differences caused by the loss of membrane‐bound thrombomodulin from endothelial cells. (A) A schematic showing the effect of the *THBD* missense variant Pro496Argfs*10 on the TM protein. There is loss of the transmembrane part of the TM protein, causing both the release of the TM into the circulation (as soluble TM, sTM) and reduced quantities on the endothelial cell surface. (A) was created with BioRender.com. (B) Representative flow cytometry histograms of WT‐TM (*n* = 5 cell lines, 2 repeats each) and MT‐TM (*n* = 2 cell lines, 2 repeats each) ECFCs stained for surface TM with (Bi) proportions of TM^+^ (CD141+) cells (Bii) and mean fluorescence intensities (MFI). (C) Levels of sTM in the cell culture supernatants of WT‐TM (*n* = 5, 2 repeats each) and MT‐TM (*n* = 2, 2 repeats each) ECFCs, showing a nearly 10‐fold greater sTM in the MT‐TM supernatants. (D) Clot formation and lysis on the surface of WT‐TM (blue) and MT‐TM (red) ECFCs showing times to 50% clot lysis (CLT_50_). (Di) Pooled normal plasma alone (PNP); (Dii) TAFI‐deficient plasma. CLT_50_ histograms, dotted lines denote normal ranges of the plasmas in nude wells. (E) Thrombin generation (ETP) on the surface of WT‐TM (blue) and MT‐TM (red) ECFCs. All results in the presence of 1pM tissue factor (Stago reagent, PPP LOW). (Bi) PNP alone; (Bii) date of activation of Protein C on the surface of WT‐TM (blue) and MT‐TM (red) ECFCs. Blank wells shown in black. (Biii) Protein C deficient plasma (PC‐DP). Black lines denote results from blank wells, and amalgamated mean curves shown. Dotted lines denote normal ranges of the two plasmas in blank wells. Blank well results were at least *n* = 20. Data shown are median, IQR. Mann–Whitney tests were used for comparison. *p* < 0.05 was considered statistically significant. *p < 0.05; **p < 0.01; ***p < 0.001; ns, non‐significant. CLT_50_, time to 50% clot lysis; C‐tail, C‐terminus tail region; ECFC, endothelial colony forming cell; EGF, epidermal growth factor‐like domain; ETP, endogenous thrombin potential; MFI, mean fluorescence intensity; MT‐TM, mutant type thrombomodulin; ST, O‐glycosylation site‐rich domain; *THBD*, thrombomodulin gene; sTM, soluble thrombomodulin; TM, thrombomodulin; WT‐TM, wild‐type thrombomodulin.

Figure 1A describes the specific *THBD* variant in our patients.^15^, ^16^ We confirm that ECFCs harvested from patients with a heterozygote *THBD* variant (MT‐TM), when compared to healthy control ECFCs (WT‐TM), had significantly reduced surface expression of TM, and released greater quantities of TM into supernatants. Flow cytometry confirmed a lower proportion of TM+ cells within MT‐TM populations: median 69.2% positivity (IQR: 61.7%–72.0%) vs. 86.2% (IQR: 77.4%–98.4%), *p* = 0.016 (Figure 1Bi) and lower immunofluorescent intensity for CD141 (TM), reduced by 2–3‐fold: 2257 (IQR: 2131–2456) vs. 6789 (IQR: 4260–9484), *p* = 0.016, MT‐TM and WT‐TM (Figure 1Bii). There was approximately 10‐fold greater sTM in the supernatant taken from MT‐TM ECFCs: 695 (IQR: 579–809) vs. WT‐TM ECFCs 75 pg/mL (IQR: 37–141); *p* = 0.002, Figure 1C.

#### 3.1.1 Clot lysis

Unstimulated MT‐TM ECFC surfaces conferred less inhibition of fibrinolysis than healthy unstimulated WT‐TM ECFCs: median time to 50% clot lysis (CLT50) 85 (IQR: 81–108) vs. 105 mins (IQR: 91–121), *p* = 0.0002. (Figure 1Di). We confirmed this effect was due to lower surface density of TM by repeating experiments using TAFI‐DP (Figure 1Dii). (TM can activate TAFI, which in turn slows clot lysis). CLT50 shortened using TAFI‐DP and the difference between MT‐TM and WT‐TM was abolished.

#### 3.1.2 Thrombin generation

Endogenous thrombin potential (ETP) was lower on the surface of MT‐TM ECFCs, median 589 (IQR: 423–1040) vs. 998 nM (IQR: 707–1771), *p* = 0.047 (Figure 1Ei). (TM activates protein C which in turn inactivates FVa and FVIIIa, thereby reducing thrombin generating capacity). In keeping with this, MT‐TM ECFC surfaces were less able to activate PC by nearly 3‐fold, *p* < 0.0001 (Figure 1Eii). Conducting TG using protein C deficient plasma (PC‐DP), there was, as expected, a loss of the inhibitory effect of ECFC surfaces on TG. ETP increased and was no difference between MT‐ and WT‐TM cell types (Figure 1Eiii). Together these findings show that MT‐TM surfaces, with low TM density, are more permissive of fibrinolysis and are better able to support thrombin generation in unstimulated conditions.

### 3.2 Thrombomodulin is lost from endothelial cell surfaces when WT‐TM ECFCs are exposed to stimulating trauma conditions increasing fibrinolytic and thrombin generating capacity

**FIGURE 2:**
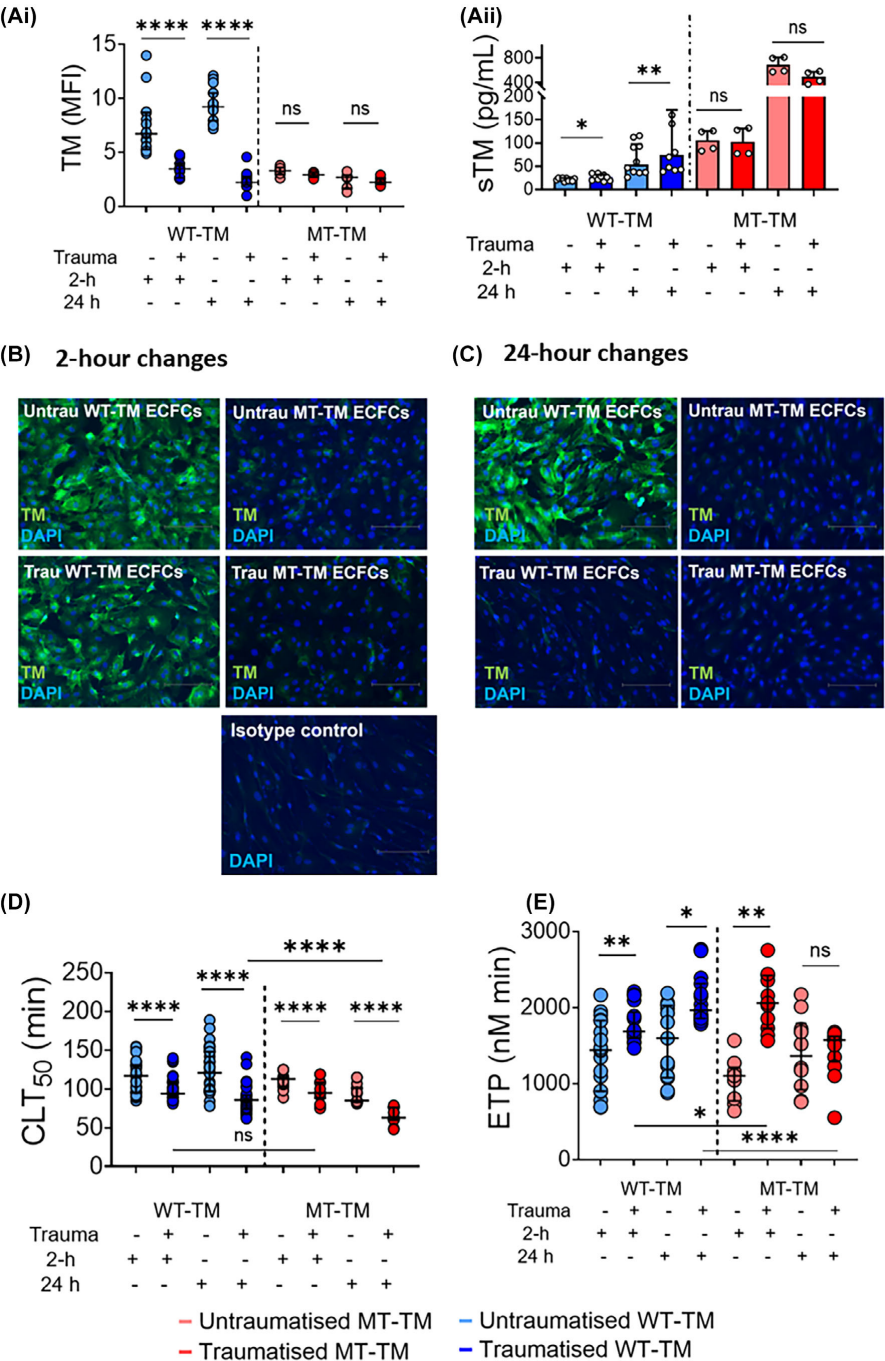
Trauma conditions lead to loss of membrane bound thrombomodulin. (A) (i) WT‐TM ECFCs and MT‐TM ECFCs were stained for membrane bound thrombomodulin (TM), after exposure to trauma conditions at 2‐ and 24‐h. Data are normalized mean fluorescence intensities (MFI). (ii) Levels of soluble TM (sTM) in the cell culture supernatants of WT‐TM and MT‐TM ECFCs after 2‐ and 24‐h exposure to trauma conditions. (B, C) Representative immunofluorescence images of WT‐TM and MT‐TM ECFCs stained for TM after (B) 2‐h of trauma conditions (C) 24‐h of trauma conditions. Staining shows DAPI, in blue and TM in green. Scale bar: 100 μm. (D) Clot formation and lysis curves on the surfaces of WT‐TM (blue) and MT‐TM (red) ECFCs, after 2 and 24 h, with median (IQR) times to 50% clot lysis (CLT_50_). Pale blue or pale red are unstimulated ECFCs and dark blue/red are stimulated ECFCs. (E) Endogenous thrombin potential (ETP) on the surface of WT‐TM (blue) and MT‐TM (red) (colors as per D), median and IQR. Comparisons made using Mann–Whitney test for unpaired data and Wilcoxon for paired data. All experiments were repeated using WT‐TM (blue, *n* = 5 cell lines, 2 replicates each) and MT‐TM (red, *n* = 2 cell lines, 2 replicates each). *p* < 0.05 considered significant. *p < 0.05; **p < 0.01; ****p < 0.0001; ns, non‐significant. ECFC, endothelial colony forming cell; ETP, endogenous thrombin potential; MFI, mean fluorescence intensity; MT, mutant type ECFC; sTM, soluble thrombomodulin; TM, thrombomodulin; WT, wild type ECFC.

Exposing WT‐TM cells to trauma conditions for up to 24‐h led to significant loss of membrane‐bound TM, at both 2‐ and 24‐h (Figure 2Ai, B, C). This change was accompanied by significant increases in sTM supernatant levels (Figure 2Aii). Trauma stimuli did not alter the amount of TM on the surfaces of MT‐TM cells, nor did the supernatant sTM level rise appreciably (Figure 2Aii). The amount of membrane‐bound TM on WT‐TM ECFC surfaces, after 2‐ and 24‐h stimulation, fell to the same level as MT‐TM ECFCs (*p* = NS, Figure 2Ai).

Looking at the hemostatic effect of the loss of TM from WT‐TM ECFC surfaces, our next experiments showed that, as might be predicted, clot lysis became quicker (Figure 2D), *p* < 0.0001, and thrombin generating capacity increased (*p* = 0.008 at 2 h, *p* = 0.03 at 24 h), Figure 2E. The increase in ETP was mirrored by a reduction in aPC activation, data not shown. These effects might be hypothesized to be solely due to the loss of TM from the ECFC surfaces following stimulation. However, clot lysis on the MT‐TM ECFC surfaces also shortened at both timepoints, with no concomitant loss of membrane‐bound TM. Similarly, ETP rose at 2‐h of stimulation (unchanged at 24‐h) on the surfaces of MT‐TM ECFCs (Figure 2E). Furthermore, there was no change to aPC activation on MT‐TM cells after stimulation, confirming no loss of membrane‐TM (data not shown). These results suggest that other hemostatic proteins are playing an important role on the EC surface and that TM, although important, is not the only effector of TIC.

### 3.3 Overall hemostatic potential at the endothelial‐coagulation interface is dictated by the constituents of trauma patient plasma, not the ECFC surface

**FIGURE 3:**
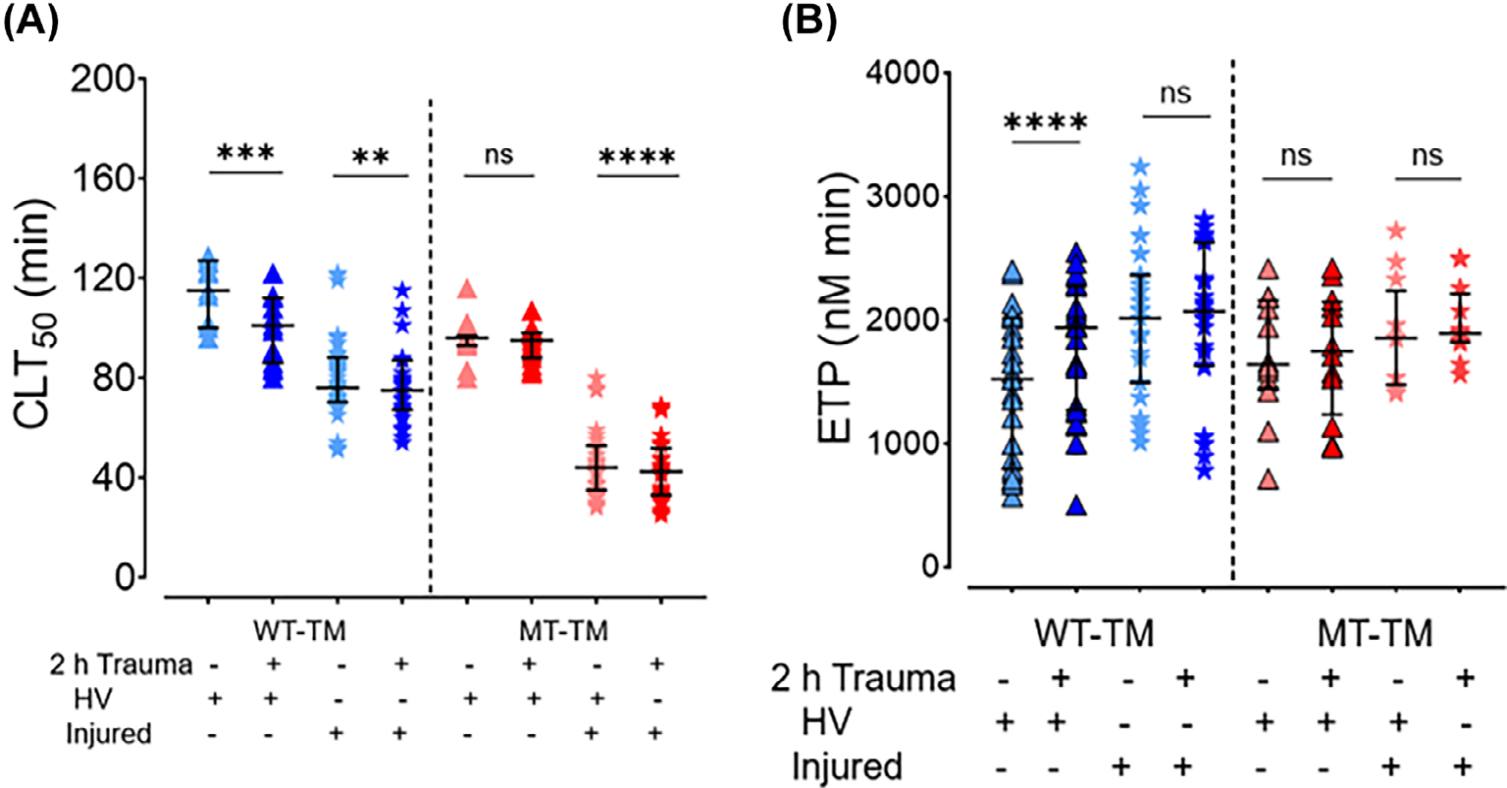
Plasma from trauma patients behaves differently on ECFCs when compared to plasma from healthy volunteers. (A) Clot formation and lysis on the surface of WT‐TM (blue) and MT‐TM (red) ECFCs. Pale blue or red lines denote untraumatized ECFCs; bright blue or red denote traumatized ECFCs. (Ai) Times to 50% clot lysis for each experimental group: Blue = WT‐TM, red = MT‐TM; pale blue/red = untraumatized ECFCs; bright blue/red = traumatized ECFCs; triangles = healthy volunteer plasma; stars = trauma patient plasma. Median, IQR. (B) Endogenous thrombin potential on the surface of WT‐TM (blue) and MT‐TM (red) ECFCs. All results performed with Microparticle reagent, 4 μM PPL. Experimental groups: Blue = WT‐TM, red = MT‐TM; pale blue/red = untraumatized ECFCs; bright blue/red = traumatized ECFCs; triangles = healthy volunteer plasma; stars = trauma patient plasma. Median and IQR. All experiments were repeated using WT‐TM (blue, *N* = 1 cell line, 3 replicates each) and MT‐TM (red, *N* = 1 cell line, 3 replicates each), 10 HV controls and 15 trauma patients. *p* < 0.05 considered significant. Comparisons made using Mann–Whitney test for unpaired data and Wilcoxon for paired data. ****p < 0.0001; ns, non‐significant. CLT50, time to 50% clot lysis; ECFC, endothelial colony forming cell; ETP, endogenous thrombin potential; HV, healthy volunteer; MT, mutant type ECFC; WT, wild type ECFC. The terms “traumatized” or “untraumatized” refer to the exposure (or lack) of the ECFCs to a “trauma cocktail” of cytokines, DAMPs, catecholamine, and hypoxia.^20^ In contrast, “trauma plasma” refers to plasma samples drawn from trauma patients at admission to hospital.

We used plasma samples drawn within 2 h of injury from trauma patients (*n* = 15) (Table 1) and exposed it to WT‐TM and MT‐TM surfaces, both unstimulated and stimulated (for 2‐h). Broadly, our results show that, as expected,^8^ trauma plasma lysed significantly more quickly than healthy volunteer (HV) plasma (*p* < 0.0001) (Figure 3A) in all experimental conditions.

**TABLE 1:** Baseline clinical characteristics of the trauma patients.

	Trauma patients *n* = 15
Age, mean (SD)	40.4 (20)
ISS, median	14 (IQR: 4–25, range 1–29)
Male, *n* (%)	12 (80%)
Blunt injury, *n* (%)	15 (100%)
GCS, median	15 (IQR: 14–15, range 6–15)
Time from injury to ED, minutes	87 (SD 21, range 19–120)
SBP, mmHg, mean (SD)	133 (SD 26, range 80–170)
HR, bpm, mean (SD)	90 (SD 23; range 58–135)
In receipt of TXA pre‐admission, *n* (%)	2 (13%)
Base excess, mEq/mol, median (IQR)	0.2 (−2.1–2.0)
Pre‐hospital	
Crystalloid, mL, median	0 (IQR: 0–0, range 0–1000)
PRBC, units	0
FFP, units	0
Bloods at admission	
Hb, g/L, mean (SD)	144 (14)
Platelet count, ×10^−9^/L, mean (SD)	242 (69)
APTT, s, median (IQR)	28.9 (22.2–31)
INR, ratio, median (IQR)	1.0 (0.9–1.0)
Clauss Fg, g/L, median (IQR)	2.4 (2.0–3.0)
D‐dimer, ng/mL, median (IQR)	7445 (2199–19,400)

Abbreviations: APTT, activated partial thromboplastin time; CA5, clot amplitude at 5 min; ED, emergency department; FFP, fresh frozen plasma; Fg, fibrinogen; GCS, Glasgow Coma Score; Hb, hemoglobin; HR, heart rate; INR, international normalized ratio; ISS, injury severity score; ML, maximal lysis; PRBC, packed red blood cells; PT, prothrombin time; SBP, systolic blood pressure; TBI, traumatic brain injury; TXA, tranexamic acid.

HV plasma, when exposed to traumatized WT‐TM surfaces, responded with shorter CLT50 compared to when exposed to non‐traumatized WT‐TM cells (*p* = 0.0008, Wilcoxon), and became the same as “HV plasma + (un)‐traumatized MT‐TM cells” (*p* = NS) (Figure 3A). This can be explained by the loss of TM from traumatized WT‐TM surfaces and confirmed that this loss can have an important effect on overall endothelial‐coagulation fibrinolytic potential.

In contrast, the pattern of CLT50 response differed when trauma plasma was tested (Figure 3A). Shortening of CLT50 was seen with WT‐TM traumatization, but here, there was a pronounced additional shortening in CLT50 on MT‐TM cell surfaces (*p* < 0.0001) suggesting that the TM density on the ECFC surface was no longer exerting a dominant lytic effect and was being masked by constituents of the trauma plasma. On MT‐TM cells, where there was no loss of TM, the traumatized MT‐TM surface plus trauma plasma supported quicker lysis. Concentrations of coagulation factors in the plasmas were compared (Table 2). Of those affecting fibrinolysis, sTM levels were higher in the trauma samples whereas t‐PA and PAI‐1 were no different.

**TABLE 2:** Hemostatic proteins in plasma from trauma patients within 2 h of admission.

Result	Trauma patients (*n* = 15)	Healthy volunteers (*n* = 20)	*p*‐Value
Soluble thrombomodulin (ng/mL)	10.9 (8.5–11.8)	7.2 (6.9–9.2)	0.04
Fibrinogen antigen (g/L)	2.1 (1.7–3.0)	2.7 (1.8–4.7)	0.34
FV (IU/mL)	0.72 (0.58–0.90)	0.85 (0.7–1.04)	0.17
FVIII (IU/mL)	2.6 (2.1–4.0)	1.2 (0.9–1.4)	0.01
PAP (ng/mL)	11,370 (1083–20,566)	698 (674–2414)	0.12
PAI‐1 (ng/mL)	4.3 (2.8–5.5)	2.2 (1.5–2.8)	0.17
tPA (ng/mL)	2.5 (1.3–4.1)	2.2 (0.3–3.0)	0.29
aPC (ng/mL)	3.1 (1.3–4.1)	1.6 (1.3–3.2)	0.70

*Note*: All results given as median value and inter‐quartile range and cohorts compared using Mann–Whitney test.

Abbreviations: aPC, activated protein C; F, factor; PAI‐1, plasminogen activator inhibitor‐1; PAP, plasmin anti‐plasmin; tPA, tissue plasminogen activator.

Broadly, trauma plasma supported quicker TG with shorter lag times and times to peak, and greater peak thrombin, when compared to HV plasma on ECFC surfaces (Figure 3B).^8^ The addition of a traumatized ECFC surface led to significantly shorter lag and times to peak, data not shown; however, ETP values were no different comparing either HV or trauma plasmas across untraumatized/traumatized ECFCs (Figure 3B), with the exception that ETP rose on traumatized WT‐TM cells in the presence of HV plasma. Greater FVIII concentrations (*p* = 0.02) were found in trauma plasma, which may explain greater peak thrombin values (Table 2).

## 4 DISCUSSION

Trauma‐induced coagulopathy and endotheliopathy are tightly linked to each other and to poor clinical outcomes.^11^ The effects of trauma‐induced endotheliopathy on hemostasis, both at the endothelial cell surface and in combination with the well‐defined changes in trauma patient plasma, has not been investigated. Here, we focus on the role of thrombomodulin at the endothelial‐coagulation interface by characterizing hemostatic capacity on ECFCs from healthy individuals and patients with an inherited *THBD* bleeding disorder, and by using experimental trauma conditions.

Previous work from our lab has demonstrated that ECFCs express functional TM and regulate hemostasis similarly to other vascular ECs, supporting our choice of ECFCs for these experiments.^20^ Furthermore, we have shown that stimulating ECs with a physiologically relevant “trauma cocktail,” recapitulates trauma‐induced endotheliopathy^20^ and that the EC hemostatic changes over a 24‐h period are multiple and complex. In this report, by using ECFCs from patients with a heterozygote *THBD* variant (MT‐TM), we were able to focus on the role that TM plays within this complex condition.

We confirm that the density of membrane‐bound TM is reduced on ECFC surfaces carrying the *THBD* variant and that TM is released in excess from these cells (Figure 2).^15^, ^16^ Using an optimized experimental plasma system, we were able to delineate the effect of reduced membrane‐TM on global hemostatic capacity of ECFCs (Figure 3) and confirmed that the surfaces of MT‐TM ECFCs were more permissive of fibrinolysis and had a greater capacity to support thrombin generation, when in their unstimulated state compared to WT‐TM cells. These findings are complementary to our previous data showing that plasma from *THBD* variant patients had both hypofibrinolytic and lower thrombin generating capacity, caused by very high sTM levels.^15^ Patients with *THBD* variants bleed only at times of injury (e.g., surgery), such that the effects of the pro‐fibrinolytic EC surface, and the low thrombin generating ability of the plasma, appear to phenotypically predominate at the site of injury. When uninjured, for example, without local TF exposure, these same ECs support greater TG than the norm, perhaps providing insight into the mild hemorrhagic nature of this condition.

When ECFCs were exposed to trauma stimuli, we show what has only previously been intimated,^8^, ^9^, ^10^, ^11^ that TM is lost from the surface of endothelial cells, and in our experiments, reduces the TM surface density to that of MT‐TM ECFCs (Figure 2). We have previously found, using RNA sequencing analysis, that there is an increased (at 2‐h), and then reduced (24‐h), expression of *THBD* mRNA in trauma‐stimulated ECFCs^20^ which further supports the hypothesis that the predominant source of increased sTM in trauma patient's plasma is from endothelial cell shedding, and which our data show takes place rapidly, within 2‐h of injury.

These experiments were designed to focus on hemostatic changes effected by TM concentration. We show that trauma stimuli conditions lead to quicker fibrinolysis on both WT‐ and MT‐ECFC surfaces, whether a discernible loss of membrane‐TM is seen. This confirms that TM is not the sole fibrinolytically active protein that plays an important functional role at the cell surface after injury. It might be assumed, but requires further investigation, that since the CL assay we used is sensitive to PAI‐1, t‐PA, and TAFI (and since there are no natural inhibitors of TAFI), t‐PA may be an important effector.

We also show that trauma stimuli increase ECFC surface thrombin generating potential, particularly in the presence of exogenous TF. TF activates thrombin via the TF‐FVIIa pathway which is rapidly inhibited by TFPI. Circulating TFPI levels rise after endothelial injury^30^ and we speculate that following injury, any membrane loss of TFPI combined with lower membrane‐TM will synergistically potentiate thrombin generation. At the sites of direct vessel injury, this will be beneficial, supporting hemostasis. Ongoing loss of membrane‐TM at 24‐h may contribute to an increased thrombotic risk and may contribute to the known association between severe injury and multi‐organ failure, kidney and acute lung injury.^11^, ^31^

Finally, we differentiate the contribution of the endothelium and the plasma to overall hemostatic capacity. Our clot lysis results show that the TM loss at the WT‐TM surface following stimulation is detected using HV plasma and appears to dictate the resultant clot lysis times. The same is not evident with trauma plasma, where quicker lysis was seen on both traumatized surfaces suggesting the coagulation abnormalities in trauma plasma mask EC‐membrane changes. Similarly, the thrombin generation experiments showed little change to ETP when using trauma plasma, again in contrast to HV plasma. Taken together, this means that membrane‐TM has little overall effect on the global hemostatic capacity at the endothelial‐coagulation interface during early TIC, and that circulating plasma components exert the most impact. The levels of coagulation factors (Table 2) are no different, except for FVIII, between the trauma and HV cohorts. The higher FVIII level will likely counterbalance the loss of surface TM in the thrombin generation assays. In addition, the authors speculate that there will likely be a high concentration of procoagulant microvesicles in the trauma plasma leading to greater thrombin generating capacity.^32^ Another possible explanation, which warrants future investigation, is that microvesicles may also be rich in annexin II (a co‐receptor for t‐PA and plasminogen),^33^ which would shorten clot lysis times.

There are limitations to this work. Due to the rarity of the condition, we were able to culture only two cell lines from *THBD* patients. ECFCs are heterogenous and other hemostatically active ECFC‐surface proteins will not be standardized, and some of our findings may reflect this heterogeneity.^34^ Our focus in this study was on TM and the preliminary evaluation of our two *THBD* cell lines (Figure S2) showed that despite their heterogeneity, the global hemostatic tests we optimized for this study were sensitive to the ECFC surface TM concentration. These experiments have been performed using a static model, using only plasma samples and future work will focus on optimizing the model under blood flow, as well as using whole blood samples to include the contributions of red cells and platelets. The addition of shear forces, for example, using venous and arterial blood flow rates, will be vital to the development of a more physiological model, since endothelial cell stretch is known to upregulate thrombomodulin expression on ECs.^35^ The numbers of trauma patient samples we have used is small, and none of the patients had significant hypocoagulable TIC, which will limit the applicability of these results to all trauma patient groups.

To conclude, we show that TM is shed from trauma‐stimulated ECFCs, permitting quicker fibrinolysis and greater thrombin generation. However, these effects are masked by the hemostatic changes in the plasma of trauma patients which exert a more dominant influence, in these experimental systems, on global measures of hemostasis.

## AUTHOR CONTRIBUTIONS

**Jeries Abu‐Hanna** performed and analyzed experiments, drafted and revised the manuscript; **Gael B. Morrow** designed experiments and revised the manuscript; **Gang Xu** performed experiments; **Lewis Timms** performed experiments; **Naveed Akbar** supervised experiments and revised the manuscript; **Michael Laffan** supervised experimental design and revised the manuscript; **Robin P. Choudhury** supervised experimental design and revised the manuscript; **Nicola Curry** conceived the project, supervised experimental design, drafted and revised the manuscript.

## CONFLICT OF INTEREST STATEMENT

The authors have disclosed no conflicts of interest.

## Supporting information


**Figure S1:** ECFCs exhibit an endothelial phenotype comparable to HUVECs.
**Figure S2:** MT‐TM ECFCs are more permissive of fibrinolysis and support greater thrombin generation than the WT‐TM ECFCs.
**Table S1:** Demographic data of the healthy volunteers and patients from whom the ECFCs were isolated.
**Table S2:** Antibodies and isotype controls used in flow cytometry. *Per 500,000 cells.
**Table S3:** Primary and secondary antibodies used in immunofluorescence.
**Data S1:** Supplemental method.

## Data Availability

The data that support the findings of this study are available from the corresponding author upon reasonable request.
