# Supplementary material for: Endothelial thrombomodulin—Its role in trauma‐induced coagulopathy
Source: Transfusion. 2026 Jan 29;66(Suppl 1):S246–56. doi: 10.1111/trf.70089 (PMC13184436; doi:10.1111/trf.70089)
Supplement: Supplementary file 1 — Figure S1: ECFCs exhibit an endothelial phenotype comparable to HUVECs. Figure S2: MT‐TM ECFCs are more permissive of fibrinolysis and support greater thrombin generation than the WT‐TM ECFCs. Table S1: Demographic data of the healthy volunteers and patients from whom the ECFCs were isolated. Table S2: Antibodies and isotype controls used in flow cytometry. *Per 500,000 cells. Table S3: Primary and secondary antibodies used in immunofluorescence. Data S1: Supplemental method. [file TRF-66-S246-s001.docx]

**Supplementary Material.**

1. **Supplemental methods**

**Cell culture.** ECFC cultures were established as previously described^1,2^. Blood was drawn into citrated vacutainers from 5 donors with wildtype TM (WT-TM) and 2 patients heterozygous for the TM mutation p.Pro496Argfs*10 (MT-TM) (Supplementary table 1). Bloods were diluted with an equal volume of PBS (Gibco), gently layered on 15 ml of Ficoll-Paque Plus (GE Healthcare) and centrifuged at 1000*g* for 20 min with both the accelerator and decelerator turned off. The buffy coat layer was aspirated, mixed with PBS, and centrifuged at 540*g* for 7 min to pellet the peripheral blood mononuclear cells (PBMCs). PBMCs were then resuspended in endothelial cell growth medium 2 (EGM2; Lonza) supplemented with 20% foetal bovine serum (FBS; Gibco) and centrifuged at 540*g* for 10 min. PBMCs were resuspended at 700,000 cells/ml and seeded in 6-well plates precoated with rat tail collagen type I (Corning) at 5 µg/cm^2^ in 0.02 M acetic acid (Merck Millipore) for 1.5 hr at 37°C to allow for collagen crosslinking. First media change was performed after 48 hr and then every 3 days until ECFC colonies appeared between 14 and 28 days. Once the colonies grew to a diameter > 1 mm, they were detached with 0.25% trypsin-EDTA (Gibco), expanded in T25 flasks and cryopreserved in FBS containing 10% (v/v) DMSO. For clot lysis, thrombin generation and protein C activation assays, ECFCs were seeded in 96-well plates at 10,000 cells/well and incubated for 48 hr. Prior to performing the assays, ECFCs were washed twice with PBS. For protein extraction, ECFCs were seeded in 6-well plates at 100,000 cells/well and grown to confluence.

***In vitro* traumatisation of ECs.** ECFCs were treated with a cocktail of trauma-related factors at concentrations within ranges measured in trauma patients, including 1 nM epinephrine^3-5^ (Sigma-Aldrich), 0.1 ng/ml TNFα^6^ (R&D Systems), 0.1 ng/ml IL-6^7,8^ (R&D Systems), 500 ng/ml HMGB1^9,10^ (Abcam) and 10 μM H_2_O_2_,^11^ and incubated for 2 and 24 hr in a hypoxia chamber with 1% O_2_. HUVECs or ECFCs were also treated with buffers used to reconstitute the recombinant proteins (i.e., PBS) and incubated for 2 and 24 hr in a standard, humidified CO_2_ incubator with ~20% O_2_.

**Flow cytometry.** Cultured ECFCs were detached using 0.25% trypsin-EDTA and resuspended in Cell Staining Buffer (Biolegend) at 5,000,000 cells/ml. 500,000 cells were pre-incubated with Human TruStain FcX (Biolegend) for 10 min at room temperature (RT) to block the Fc receptors. Cells were then incubated with fluorophore-conjugated antibodies or isotype controls (Supplementary Table 2) for 20 min on ice in the dark. Cells were washed twice with Cell Staining Buffer and centrifugation at 350*g* for 5 min and stained with live/dead dye 7-AAD (Biolegend) for 5 min on ice in the dark. Flow cytometry was performed on the BD LSRFortessa X20 Cell Analyser (BD Biosciences), capturing 10,000 events, and data were analysed using the FlowJo software (v10.10). Following doublet and dead cell exclusion, cells were assessed for surface expression of CD45, CD34, CD31, CD144 (VE-cadherin), CD309 (VEGFR2) and CD141 (TM).

**Immunocytochemistry.** Cells were seeded at a density of 15,000 cells per chamber in Nunc Lab-Tek 8-chamber slides (ThermoFisher Scientific). After reaching confluence, cells were washed thrice with PBS and fixed in 4% paraformaldehyde in PBS (Santa Cruz Biotechnology) for 15 min. Fixed cells were washed thrice with PBS to remove excess PFA and permeabilised with PBS with 0.1% Triton X-100 for 5 min. Permeabilised cells were washed thrice with PBS and blocked with 10% Normal Goat Serum (derived from the same species as the secondary antibody) in PBS (ThermoFisher Scientific) for 1 hr at room temperature. Cells were incubated with primary antibodies (Supplementary Table 2) in Antibody Diluent (Dako) at 4°C overnight. After incubation, cells were washed three times with PBS and incubated with fluorescent secondary antibodies (Supplementary Table 3) and Alexa Fluor 647-Phalloidin (Invitrogen; 1: 300) in Antibody Diluent in the dark for 1 hr at room temperature. Cells were washed three times with PBS and mounted with ProLong Gold Antifade Mountant containing the nuclear stain DAPI (Invitrogen). Cells were imaged using a Leica TCS SP8 confocal laser scanning microscope.

**Enzyme-linked immunosorbent assay (ELISA).** Cell culture supernatants were collected from HUVECs and ECFCs and centrifuged at 1000*g* for 10 min to remove cell debris. ELISAs for sTM (undiluted; ab214029; Abcam), PAI-1 (dilution: 1 in 200; ab108891; Abcam) and tPA (dilution: 1 in 10; ab190812; Abcam) were performed following the manufacturer’s instructions.

**Clot lysis assay.** Clot lysis was assayed as previously described^12^. Mixtures of 30% (v/v) CRYOcheck pooled normal plasma (PNP; PrecisionBiologic), 16 µM Rox Phospholipid TGT (composed of 42% phosphatidyl choline, 28% phosphatidyl serine and 30% sphingomyelin; Rossix) and 50 pM tPA (Sigma-Aldrich) in Tris-buffered saline (TBS; 10 mM Tris, 140 mM NaCl, pH7.4) were added to wells devoid of cells and wells seeded with cells, referred to as nude and seeded wells, respectively. In some cases, TAFI-deficient plasma (TAFI-DP; Affinity Biologicals) was used in place of PNP. To investigate the effects of cell culture supernatants on clot lysis, PNP was supplemented with 20% (v/v) cell culture supernatant. Clotting was initiated with a mixture of 0.1 U/ml thrombin from human plasma (Sigma-Aldrich) and 10.6 mM CaCl_2_. Clot formation and lysis were monitored by measuring absorbance at 405 nm at 1-min intervals for 4 hr using a Multiskan Ascent microplate reader (ThermoScientific) and the Ascent Software (v2.6). Times to 50% lysis (CLT_50_) in min were determined using the Shiny app for clot lysis^13^.

**Thrombin generation assay.** Thrombin generation was assessed using the Calibrated Automated Thrombography (CAT) method^14^. PPP-Reagent LOW (Diagnostica Stago), containing 1 pM TF, was added to nude and seeded wells, with PNP, and the plate was incubated at 37°C for 10 min prior to the addition of FluCa (Diagnostica Stago), containing CaCl_2_ and the fluorogenic, thrombin-specific substrate Z-Gly-Gly-Arg 7-amido-4-methylcoumarin. Fluorescence was measured every 30 s for 90 min using a Fluoroskan Ascent Fluorometer (ThermoScientific) in conjunction with the Thrombinoscope software (v5.0, Thrombinoscope BV). In some cases, protein C-deficient plasma (PC-DP; Affinity Biologicals) was used in place of PNP.

**Protein C activation assay.** PC activation was assessed as previously described^15^ with some modifications. 70 nM PC from human plasma (Sigma-Aldrich) in PBS (Gibco) supplemented with 0.6 mM MgCl_2_ and 1% (w/v) BSA was added to nude and seeded wells. A mixture of 0.1 U/ml thrombin and 3 mM CaCl_2_ in PBS was then added, and the plate was incubated at 37°C for 30 min. 1 U/ml hirudin (Sigma-Aldrich) was added to inhibit thrombin and stop the reaction. 0.42 mg/ml BIOPHEN CS-21(66) (HYPHEN BioMed), a chromogenic substrate for aPC, was added and absorbance at 405 nm was measured every 30 s for 2 hr using a Multiskan Ascent microplate reader (ThermoScientific) and the Ascent Software (v2.6). PC activation rates were calculated using the Shiny app for zymogen activation^13^.

1. **Supplemental Tables**

**Supplemental Table 1:** Demographic data of the healthy volunteers and patients from whom the ECFCs were isolated.

| **ECFC** | **Age** | **Sex** | **Number of colonies** | **Time to colony appearance (days)** |
| --- | --- | --- | --- | --- |
| **Controls** | | | | |
| 1 | 49 | Female | 4 | 16 |
| 2 | 26 | Female | 3 | 17 |
| 3 | 34 | Female | 4 | 17 |
| 4 | 42 | Female | 9 | 9 |
| 5 | 28 | Female | 3 | 23 |
| **Patients** | | | | |
| 6 | 70 | Female | 5 | 22 |
| 7 | 48 | Female | 3 | 23 |

**Supplemental Table 2:** Antibodies and isotype controls used in flow cytometry. *Per 500,000 cells.

| **Antibody** | **Species** | **Volume (µl)*** | **Supplier** | **Catalogue No.** |
| --- | --- | --- | --- | --- |
| Alexa Fluor 647 anti-human CD144 (VE-cadherin) | Mouse | 2.5 | Biolegend | 348513 |
| Pacific Blue anti-human CD34 | Mouse | 2.5 | Biolegend | 343511 |
| Brilliant Violet 605 anti-human CD141 (TM) | Mouse | 2.5 | Biolegend | 344117 |
| APC/Cyanine anti-human CD45 | Mouse | 2.5 | Biolegend | 368515 |
| PE anti-human CD309 (VEGFR2) | Mouse | 2.5 | Biolegend | 393003 |
| FITC anti-human CD31 (PECAM1) | Mouse | 5 | BD Biosciences | 555445 |
| Alexa Fluor 647 Mouse IgG2a κ isotype control | Mouse | 20 | Biolegend | 400234 |
| Pacific Blue IgG1 κ isotype control | Mouse | 2.5 | Biolegend | 400131 |
| Brilliant Violet 605 IgG1 κ isotype control | Mouse | 3.75 | Biolegend | 400161 |
| APC/Cyanine 7 IgG1 κ isotype control | Mouse | 0.625 | Biolegend | 400127 |
| PE IgG1 κ isotype control | Mouse | 2.5 | Biolegend | 393003 |
| FITC IgG1 κ isotype control | Mouse | 2.5 | Biolegend | 981802 |

**Supplemental Table 3:** Primary and secondary antibodies used in immunofluorescence.

| **Antibody** | **Species** | **Dilution** | **Supplier** | **Catalogue No.** |
| --- | --- | --- | --- | --- |
| **Primary** | | | | |
| TM | Mouse | 1:500 | Abcam | ab6980 |
| CD31 | Mouse | 1:100 | Dako | M0823 |
| vWF | Rabbit | 1:200 | Dako | A0082 |
| Syndecan-1 | Rabbit | 1:200 | Abcam | ab128936 |
| TF | Rabbit | 1:100 | Abcam | ab228968 |
| TFPI | Rabbit | 1:100 | Abcam | ab180619 |
| IgG1 kappa isotype control | Mouse | 1:250 - TM  1:248 - CD31 | Invitrogen | 14-4714-82 |
| IgG polyclonal isotype control | Rabbit | 1:357 - vWF | Abcam | ab37415 |
| **Secondary** | | | | |
| Anti-mouse IgG, DyLight 488-linked | Goat | 1:250 | Invitrogen | 35502 |
| Anti-rabbit IgG, Alexa Fluor 488-linked | Goat | 1:500 | Invitrogen | A-11008 |

**Supplementary Figure 1.** ECFCs exhibit an endothelial phenotype comparable to HUVECs.


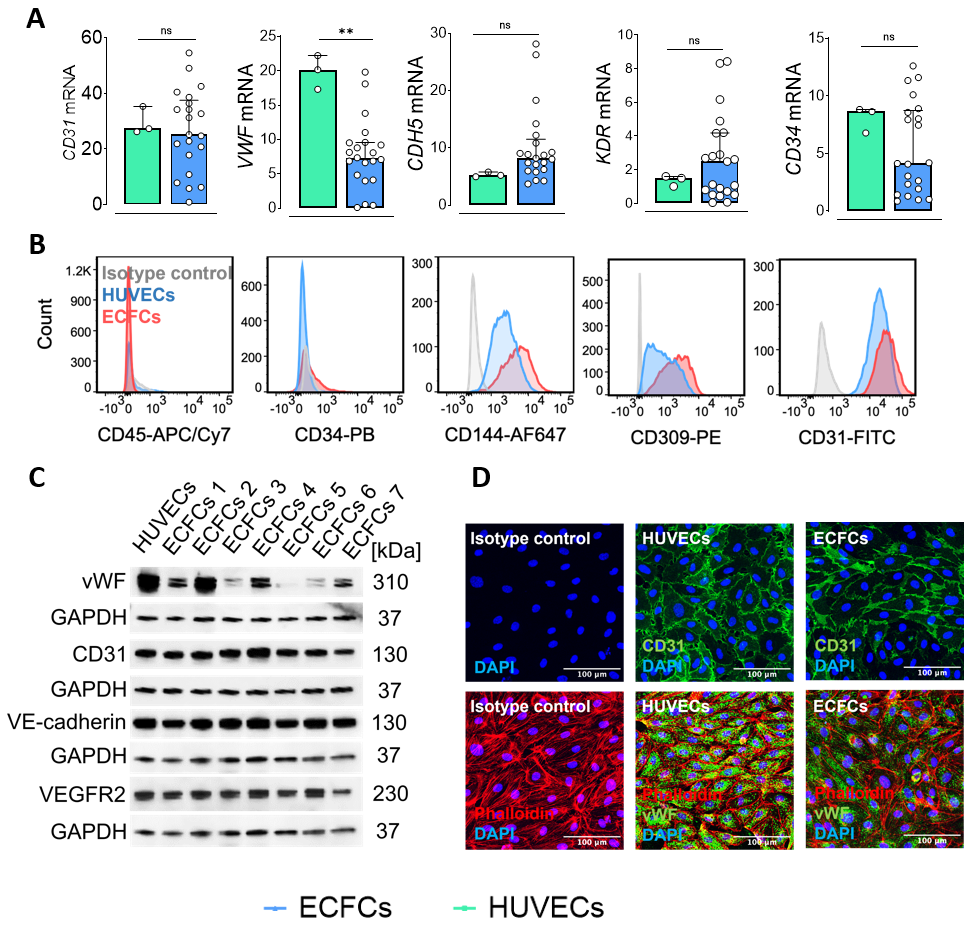
**Figure Legend:**

**(A)** Transcript levels of CD31, VWF, CDH5 and CD34 genes relative to the housekeeping genes TBP, B2M and GAPDH in HUVECs (n = 1 cell line, 3 repeats) and ECFCs (n = 7 cell lines, 3 repeats each). **(B)** Flow cytometric analysis of HUVECs and ECFCs for surface expression of CD45, CD34, CD31, CD309 (VEGFR2) and CD144 (VE-cadherin). Histograms are representative of HUVECs and ECFCs. Negativity for CD45 and CD34, and concomitant positivity for CD31, CD144 and CD309 confirm the endothelial nature of these cells. **(C)** Immunoblotting of proteins from HUVECs (n = 1) and ECFCs (n = 7) for CD31, VEGFR2, vWF and VE-cadherin. GAPDH was used as a control. **(D)** Representative images of HUVECs and ECFCs using immunofluorescent stains. Top row: cells were stained for the presence of CD31 (green) and nuclei (DAPI – in blue). Bottom row images show HUVECs and ECFCs stained for nuclei (DAPI- in blue), vWF (green), phalloidin (red) to visualise the actin cytoskeleton and reveal the cobblestone morphology that is characteristic of endothelial cells. Scale bar: 100 μm. Data are median and interquartile range, comparisons made using Mann-Whitney test. NS = not significant; * - p<0.05; ++ - p<0.01

**Supplementary Figure 2:** MT-TM ECFCs are more permissive of fibrinolysis and support greater thrombin generation than the WT-TM ECFCs.


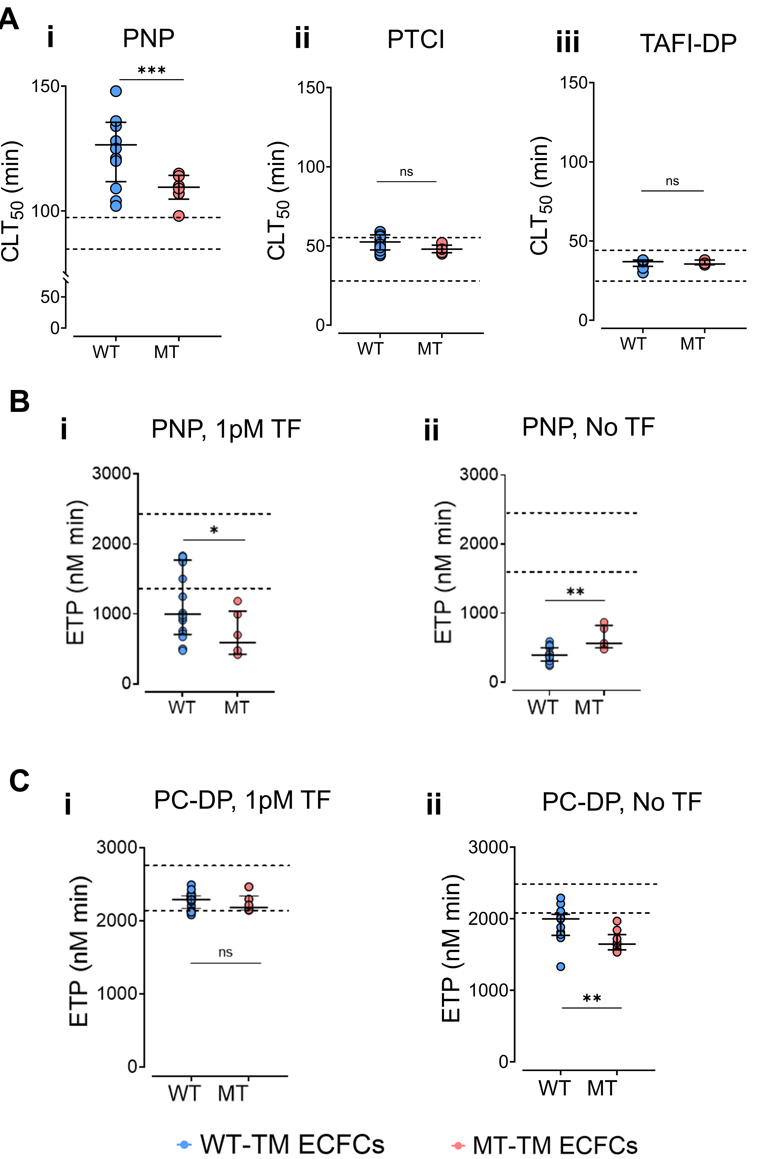


**Figure Legend:**

**(A)** Clot formation and lysis on the surface of WT-TM (blue) and MT-TM (red) ECFCs showing times to 50% clot lysis (CLT_50_). **(Ai)** Pooled normal plasma alone (PNP); **(Aii)** PNP supplemented with PTCI; **(Aiii)** TAFI-deficient plasma. CLT_50_ histograms, dotted lines denote normal ranges of the three plasmas in nude wells. **(B)** Thrombin generation on the surface of WT-TM (blue) and MT-TM (red) ECFCs in top row, with endogenous thrombin potential (ETP) below. All results in the presence of 1pM tissue factor (Stago reagent, PPP LOW). **(Bi)** PNP alone; **(Bii)** Protein C deficient plasma (PC-DP). Black lines denote results from nude wells, and amalgamated mean curves shown. Second row: ETP histograms. Dotted lines denote normal ranges of the two plasmas in nude wells. **(C)** Thrombin generation on the surface of WT-TM (blue) and MT-TM (red) ECFCs in top row, with ETP below. All results in the absence of tissue factor (Stago Microparticle reagent). **(Ci)** PNP alone; **(Cii)** Protein C deficient plasma (PC-DP). Black lines denote results from nude wells, and amalgamated mean curves shown. Second row: ETP histograms. Dotted lines denote normal ranges of the two plasmas in nude wells.

All experiments were repeated using WT-TM (blue, n = 5 cell lines, 3 replicates each) and MT-TM (red, n = 2 cell lines, 2 or 3 replicates each). Nude well results were at least n = 20. p < 0.05 considered significant. *- p <0.05; **- p< 0.01; *** - p < 0.001; ns - non-significant.

Key. CLT_50_ – time to 50% clot lysis; ECFC – endothelial colony forming cell; ETP – endogenous thrombin potential; MT- mutant type ECFC; WT – wild type ECFC

**References**

1. Martin-Ramirez J, Hofman M, van den Biggelaar M, et al. Establishment of outgrowth endothelial cells from peripheral blood. Nat Protoc 2012;7(9):1709-15, doi:10.1038/nprot.2012.093

2. Ormiston ML, Toshner MR, Kiskin FN, et al. Generation and Culture of Blood Outgrowth Endothelial Cells from Human Peripheral Blood. J Vis Exp 2015;106):e53384, doi:10.3791/53384

3. Ostrowski SR, Henriksen HH, Stensballe J, et al. Sympathoadrenal activation and endotheliopathy are drivers of hypocoagulability and hyperfibrinolysis in trauma: A prospective observational study of 404 severely injured patients. J Trauma Acute Care Surg 2017;82(2):293-301, doi:10.1097/TA.0000000000001304

4. Ostrowski SR, Johansson PI. Endothelial glycocalyx degradation induces endogenous heparinization in patients with severe injury and early traumatic coagulopathy. J Trauma Acute Care Surg 2012;73(1):60-6, doi:10.1097/TA.0b013e31825b5c10

5. Johansson PI, Stensballe J, Rasmussen LS, et al. High circulating adrenaline levels at admission predict increased mortality after trauma. J Trauma Acute Care Surg 2012;72(2):428-36, doi:10.1097/ta.0b013e31821e0f93

6. Rabinovici R, John R, Esser KM, et al. Serum tumor necrosis factor-alpha profile in trauma patients. J Trauma 1993;35(5):698-702, doi:10.1097/00005373-199311000-00008

7. Gebhard F, Pfetsch H, Steinbach G, et al. Is interleukin 6 an early marker of injury severity following major trauma in humans? Arch Surg 2000;135(3):291-5, doi:10.1001/archsurg.135.3.291

8. Okeny PK, Ongom P, Kituuka O. Serum interleukin-6 level as an early marker of injury severity in trauma patients in an urban low-income setting: a cross-sectional study. BMC Emerg Med 2015;15(22, doi:10.1186/s12873-015-0048-z

9. Peltz ED, Moore EE, Eckels PC, et al. HMGB1 is markedly elevated within 6 hours of mechanical trauma in humans. Shock 2009;32(1):17-22, doi:10.1097/shk.0b013e3181997173

10. Cohen MJ, Brohi K, Calfee CS, et al. Early release of high mobility group box nuclear protein 1 after severe trauma in humans: role of injury severity and tissue hypoperfusion. Crit Care 2009;13(6):R174, doi:10.1186/cc8152

11. van der Vliet A, Janssen-Heininger YM. Hydrogen peroxide as a damage signal in tissue injury and inflammation: murderer, mediator, or messenger? J Cell Biochem 2014;115(3):427-35, doi:10.1002/jcb.24683

12. Morrow GB, Beavis J, Harper S, et al. Characterisation of a novel thrombomodulin c.1487delC,p.(Pro496Argfs*10) variant and evaluation of therapeutic strategies to manage the rare bleeding phenotype. Thromb Res 2021;197(100-108, doi:10.1016/j.thromres.2020.11.002

13. Longstaff C, fibrinolysis so. Development of Shiny app tools to simplify and standardize the analysis of hemostasis assay data: communication from the SSC of the ISTH. J Thromb Haemost 2017;15(5):1044-1046, doi:10.1111/jth.13656

14. Hemker HC, Giesen P, Al Dieri R, et al. Calibrated automated thrombin generation measurement in clotting plasma. Pathophysiol Haemost Thromb 2003;33(1):4-15, doi:10.1159/000071636

15. Rehill AM, Leon G, McCluskey S, et al. Glycolytic reprogramming fuels myeloid cell-driven hypercoagulability. J Thromb Haemost 2024;22(2):394-409, doi:10.1016/j.jtha.2023.10.006
